# Identifying socio-ecological drivers of common cold in Bhutan: a national surveillance data analysis

**DOI:** 10.1038/s41598-022-16069-7

**Published:** 2022-07-09

**Authors:** Tsheten Tsheten, Kinley Penjor, Chachu Tshering, Archie C. A. Clements, Darren J. Gray, Kinley Wangdi

**Affiliations:** 1 National Centre for Epidemiology and Population Health, College of Health and Medicine, Australian National University, Canberra, Australia; 2 Royal Centre for Disease Control, Ministry of Health, Thimphu, Bhutan; 3 Vector-Borne Diseases Control Programme, Department of Public Health, Ministry of Health, Thimphu, Bhutan; 4 Child Health Program, Communicable Diseases Division, Department of Public Health, Ministry of Health, Thimphu, Bhutan; 5 Telethon Kids Institute, Nedlands, Australia; 6 Curtin University, Perth, Australia

**Keywords:** Climate sciences, Diseases, Health care, Risk factors

## Abstract

The common cold is a leading cause of morbidity and contributes significantly to the health costs in Bhutan. The study utilized multivariate Zero-inflated Poisson regression in a Bayesian framework to identify climatic variability and spatial and temporal patterns of the common cold in Bhutan. There were 2,480,509 notifications of common cold between 2010 and 2018. Children aged < 15 years were twice (95% credible interval [CrI] 2.2, 2.5) as likely to get common cold than adults, and males were 12.4% (95 CrI 5.5%, 18.7%) less likely to get common cold than females. A 10 mm increase in rainfall lagged one month, and each 1 °C increase of maximum temperature was associated with a 5.1% (95% CrI 4.2%, 6.1%) and 2.6% (95% CrI 2.3%, 2.8%) increase in the risk of cold respectively. An increase in elevation of 100 m and 1% increase in relative humidity lagged three months were associated with a decrease in risk of common cold by 0.1% (95% CrI 0.1%, 0.2%) and 0.3% (95% CrI 0.2%, 0.3%) respectively. Seasonality and spatial heterogeneity can partly be explained by the association of common cold to climatic variables. There was statistically significant residual clustering after accounting for covariates. The finding highlights the influence of climatic variables on common cold and suggests that prioritizing control strategies for acute respiratory infection program to subdistricts and times of the year when climatic variables are associated with common cold may be an effective strategy.

## Introduction

Common cold is the most frequent upper respiratory tract infection (URTI) in people, affecting the mucosa of the nasal cavity, nasopharynx, paranasal sinuses and larynx^1^. On an average, adults are likely to catch two to three colds per year, while children can have up to eight episodes of cold each year^2^. Common cold is a heterologous group of diseases caused predominantly by > 200 viruses belonging to different families^3,4^. Rhinoviruses are the most frequent cause of common colds (30–50%) followed by coronaviruses (10–15%) and influenza viruses (5–15%)^1,5^. Patients with common cold often have co-infections with multiple viruses^1^. Bacterial infections are rarely indicated in the aetiology of common cold^3^.

Common cold viruses are transmitted via airborne droplets or hand-to-hand contact with subsequent passage to the nostrils and eye^6^. The onset of symptoms varies considerably, according to the incubation period of different viruses. In rhinovirus infections, the incubation period is typically short (10–12 h), whereas for influenza viruses, it can be as long as a week^1^. Typically, patients present with nasal congestion, sore throat, watery eyes, sneezing, rhinorrhea, cough and general malaise^7^. Fever is rare in adults but more common in children^4^. The common cold is usually a self-limiting illness lasting for 7–10 days or no more than 3 weeks^1^. However, viral infection in some patients spreads to adjacent organs, resulting in different clinical manifestations and occasionally predisposing to bacterial invasions. These secondary bacterial invaders can lead to serious adverse health outcomes^5^. The most common bacterial complications are otitis media (~ 20%) and sinusitis (~ 0.5–2%)^1^. Some illnesses progress to pneumonia as a result of bacterial complication, or due to extension of the viral infection^1^. In addition, common cold can exacerbate pre-existing conditions such as asthma, chronic obstructive pulmonary disease, and idiopathic pulmonary fibrosis^8–11^. Common colds are managed symptomatically and there are no specific therapeutics or vaccines that are effective against all viruses.

The common cold is the most prevailing illness that persists as a major public health problem with a significant socio-economic burden. The morbidity rate of common cold in China is 34.4%^12^, while it accounts for 20–40% of the outpatient consultations in India^13^. Indonesia reported a period prevalence of 25% in 2013 for respiratory infections mostly associated with more primary healthcare visits (40–60%) than hospital visits (15–30%)^14^. The illness is associated with overcrowding in the outpatient consultation, increased medication costs and loss of active labor days or loss of productivity due to absence from work or school^15^. A highly pervasive approach of non-prescription, over-the-counter self-medication for the illness gives rise to huge financial expenditure^16^. In healthcare settings, inappropriate prescription of antibiotics for the common cold exacerbates an already increasing trend of antibiotic resistance^6^.

In Bhutan, the common cold is still considered a leading cause of morbidity, according to the Annual Health Bulletin of the Ministry of Health^17^. In 2004, 264,863 cases of common cold were recorded, while there was a reduction of cases in 2018 by 12% (231,525 cases)^17^. However, there are limited studies at the national level on the trends and spatial distribution and factors that drive common cold in the country.

The common cold is a seasonal illness and environmental factors play a crucial role in the transmission dynamics of the disease^18^. In tropical regions, the frequency of viral activity and transmission of respiratory infections are influenced by demographic characteristics and changes in temperature, rainfall, and relative humidity^19^. The incidence of common cold declines with age with children experiencing more episodes with prolonged symptoms than adults^4^. On the contrary, a study conducted in China in 2019 has reported a higher incidence in adults aged > 15 years than the children aged ≤ 15 years, indicating the difference in the influence of different demographic characteristics on common cold varies by geographical locations^20^. The female sex displays increased susceptibility with greater frequency to common cold than males^21^. Recently, a study in Japan and Germany reported a significantly higher proportion of antibiotic prescriptions for a younger and female population with common cold^22,23^. Lagged climatic variables were used to understand its effect on common cold. A study in China found that the number of common colds increased by 11.1%, 10.4%, 10.6 and 10.7% for 1 °C increase in diurnal temperature at lag 0, lag 5, lag 10 and lag 14 days respectively^20^. Another study in Finland reported a significant increase in common cold as a result of linear decrease in temperature and humidity in the preceding 2 weeks^24^. Similar study in Greece reported that general practitioner (GP) consultation for respiratory infection increased by 28% for every 10 °C decrease in minimum temperature lagged at 15 days. Further, an increase of 10 g/m^3^ in absolute humidity lagged at 12 days decreased GP consultations for respiratory infections by 47%^25^. However, a study in Vietnam found that rainfall and temperature lagged 1 week were positively associated with acute respiratory infection caused by respiratory syncytial virus (RSV)^26^. Evidence also suggest strong spatial clustering of respiratory pathogens in certain locations driven by their proximities and pattern of human activities^27^. Therefore, this study aimed to quantify the effect of climatic variables on the common cold and investigate the spatial and temporal patterns of common cold in Bhutan.

## Results

Between 2010 and 2018, 2,480,509 notifications of common cold were reported in the country. Of these notifications, the proportion of the population that was aged < 15 years was 45.9% (n = 1,139,168), while those aged ≥ 15 years comprised 54.1% (n = 1,341,341). Throughout the study period, children aged < 15 years had a higher incidence as compared to people aged ≥ 15 years (Table 1).

**Table 1:** Annual Incidence of common colds stratified by age groups in Bhutan, 2010–2018.

Year	< 15 years			≥ 15 years		
	Cases	Population	Incidence^a^	Cases	Population	Incidence^a^
2010	152,191	175,644	866.47	171,518	498,509	344.06
2011	119,167	177,588	671.03	147,942	504,025	293.52
2012	132,932	179,553	740.35	158,596	509,603	311.21
2013	121,835	181,540	671.12	149,832	515,242	290.80
2014	135,340	183,549	737.35	162,261	520,943	311.48
2015	127,975	185,580	689.59	148,213	526,707	281.40
2016	134,897	187,634	718.94	151,677	532,536	284.82
2017	107,999	189,710	569.28	126,464	538,429	234.88
2018	106,832	191,807	556.98	124,838	544,381	229.32

^a^Incidence per 1000 population.

**Figure 1:**
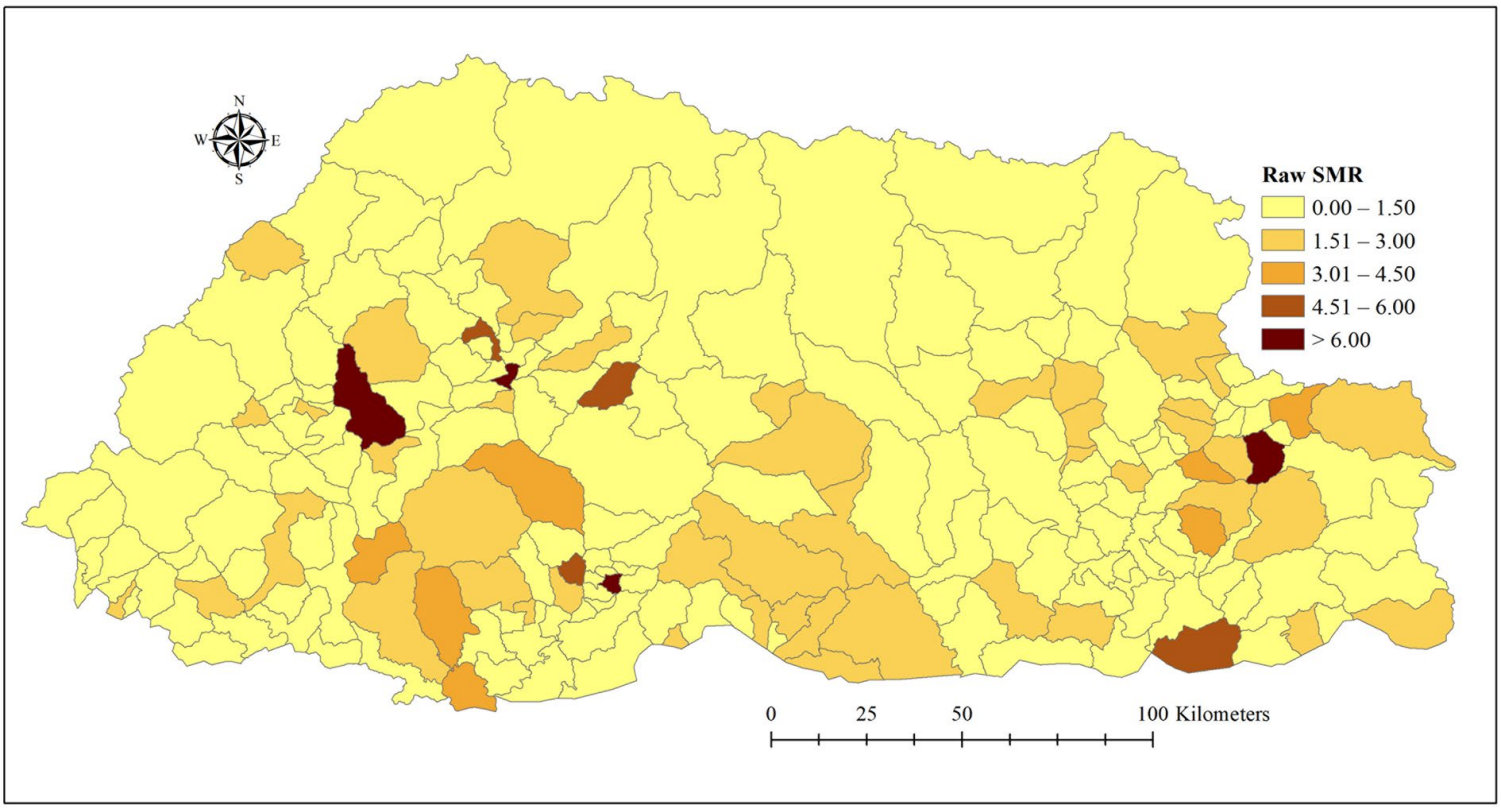
Raw standardized morbidity ratios (SMR) of the common cold by sub-districts in Bhutan, January 2010–December 2018.

The annual incidence ranged from 314.69 per 1000 inhabitants in 2018 to 480.17 in 2010. On average, 112 cases of common cold were reported every month during the study period (Table 2). A wide variation of standardized morbidity ratio (SMR) was observed across sub-districts. The highest SMR (> 6.00) were reported in Mewang (Thimphu district), Shongphu (Trashigang district), Kilkhorthang (Tsirang district) and Thedtsho (Wangdiphodrang district) subdistricts (Fig. 1).

**Table 2:** Monthly means of common colds by demographic and environmental variables in Bhutan, 2010–2018.

Exploratory variables	Mean (standard deviation)	Minimum–maximum
Cases (all combined)	112 (204.70)	0–1018
**Age groups (cases)**		
< 15 years	51.45 (105.18)	0–502
≥ 15 years	60.59 (106.14)	0–525
**Sex (cases)**		
Male	54.25 (106.86)	0–511
Female	57.78 (101.50)	0–506
Rainfall (mm)	5.20 (8.92)	0–42.52
Maximum Temperature (°C)	23.54 (5.22)	11.32–33.3
Humidity (%)	68.42 (19.04)	2.58–92.62
Altitude (m)	1969.88 (995.86)	301–4589

**Figure 2:**
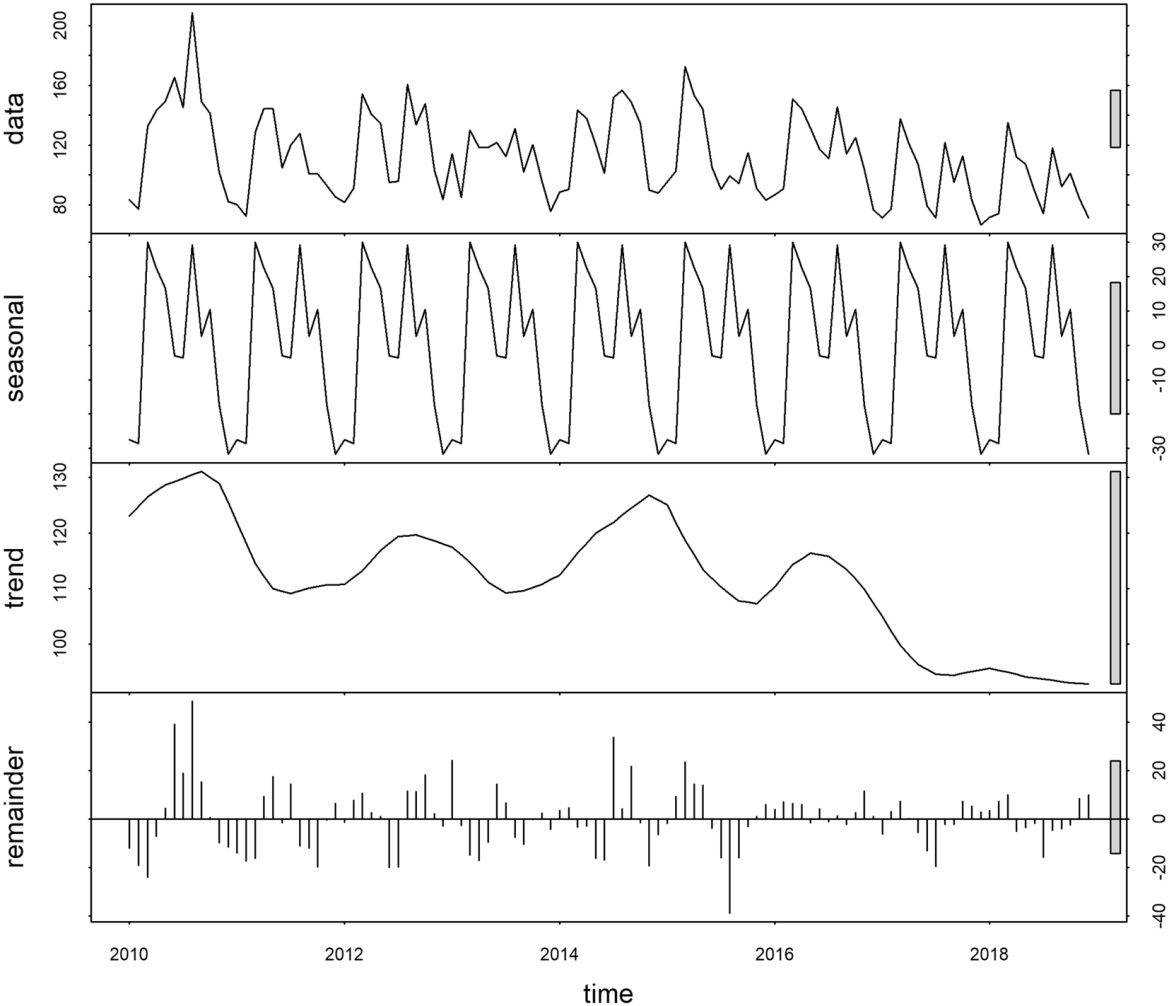
Temporal decomposition of common cold counts in Bhutan, January 2010–December 2018. Data shows the original time series, Seasonality shows the decomposed components, denoting the seasonal component, Trend shows a long-term trend component and the remainder component.

Time series analysis showed strong seasonality with spikes of case numbers before and after the summer season: February to May (early spring), and July to October (early Autumn). The inter-annual pattern exhibited an apparent decreasing trend with the highest cases observed in 2010 (Fig. 2). The monthly trend of the common colds displayed a similar presentation in both < 15 and ≥ 15 years age groups with peaks in March and August each year (Supplementary Figs. 1 and 2).

The ZIP model IV, which was the best-fitting model, revealed that children aged < 15 years were > 2 times (RR 95% CrI 2.2, 2.5) at risk of contracting common cold than the population aged > 15 years. Males were 12.4% less likely to get common cold than females (95% CrI 5.5%, 18.7%). Colds increased by 5.1% (95% CrI 4.2%, 6.1%) for every 10 mm increase of rainfall, while a 1 °C increase in temperature was associated with 2.6% increase (95% CrI 2.3%, 2.8%) in common cold cases. However, a 100 masl increase in elevation and relative humidity was associated with a decrease in risk of common colds by 0.1% (95% CrI 0.1%, 0.2%) and 0.3% (95% CrI 0.2%, 0.3%) respectively (Table 3).

**Table 3:** Relative risk and 95% credible interval from Bayesian spatial and non-spatial models of common colds in Bhutan, January 2010–December 2018.

Variable	Model I (unstructured)	Model II (structured)	Model III (convoluted)	^b^Model IV (convoluted plus spatio-temporal)
Intercept^a^	0.599 (0.463, 0.773)	− 1.346 (− 1.425, − 1.272)	0.391 (− 0.743, 1.478)	− 0.931 (− 1.770, 0.076)
Age (≥ 15 years as ref)	2.340 (2.334, 2.346)	2.342 (2.199, 2.494)	2.334 (2.262, 2.401)	2.340 (2.168, 2.530)
Sex (female as ref)	0.877 (0.874, 0.879)	0.880 (0.830, 0.930)	0.875 (0.847, 0.901)	0.876 (0.813, 0.945)
Mean monthly trend	0.996 (0.995, 0.996)	0.996 (0.995, 0.996)	0.996 (0.995, 0.996)	1.086 (1.038, 1.137)
Altitude (100 m)	0.998 (0.998, 0.999)	0.999 (0.998, 0.999)	0.998 (0.998, 0.999)	0.999 (0.998, 0.999)
Maximum temperature (°C)	1.023 (1.022, 1.023)	1.022 (1.020, 1.025)	1.022 (1.021, 1.024)	1.026 (1.023, 1.028)
Rainfall (10 mm)^c^	1.057 (1.056, 1.059)	1.058 (1.051, 1.065)	1.057 (1.054, 1.061)	1.051 (1.042, 1.061)
Relative humidity (%)^d^	0.997 (0.996, 0.997)	0.996 (0.995, 0.997)	0.996 (0.996, 0.997)	0.997 (0.997, 0.998)
Probability of extra zero	1.075 (1.073, 1.077)	1.072 (1.068, 1.077)	1.070 (1.068, 1.073)	1.038 (1.035, 1.040)
**Heterogeneity**				
Unstructured	1.073 (1.056, 1.092)	–	1.103 (1.046, 1.210)	1.205 (1.157, 1.262)
Structured (spatial)	–	1.020 (1.015, 1.025)	1.083 (1.017, 1.216)	1.123 (1.051, 1.378)
Structured (trend)	–	–	–	1.12 (1.05, 1.38)
DIC	1,175,850	1,184,090	1,181,580	1,115,850^b^

*DIC* deviation information criterion, *mm* millimetre.

^a^Coefficient; ^**b**^best-fit model; ^c^lagged 1 month; ^d^lagged 3 months.

**Figure 3:**
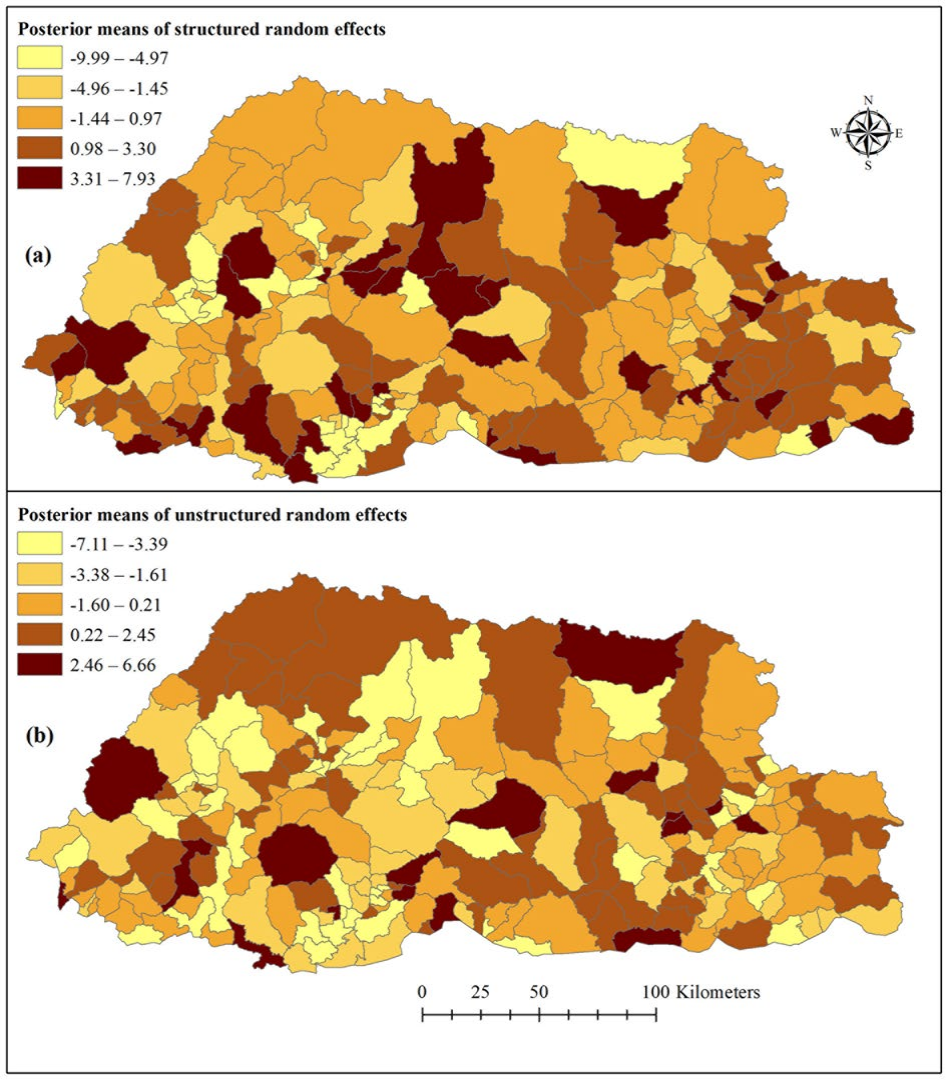
Spatial distribution of posterior means of structured (**a**) and unstructured random effects (**b**) of common cold in Bhutan, January 2010–2018.

**Figure 4:**
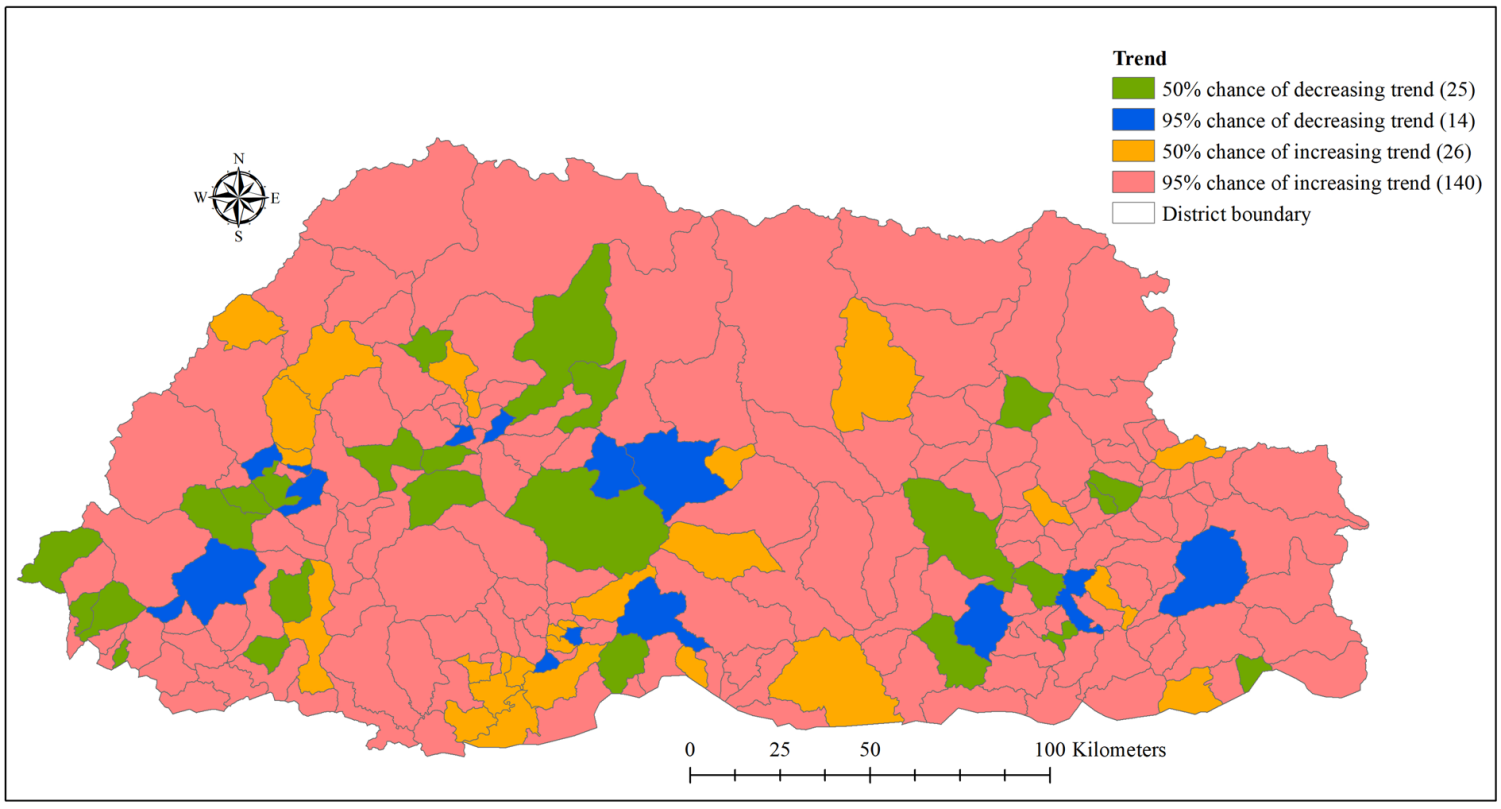
Trend analysis of common colds by sub-district in Bhutan (January 2010–December 2018) based on the spatio-temporal random effect of a Bayesian model.

There was spatial clustering after accounting for the covariates (Fig. 3). Spatial clustering was observed predominantly in the Western region of the country, where the capital Thimphu is part of it. Some sub-districts had a high probability of being above (or below) the overall mean residual risk (Supplementary Fig. 3). A higher trend than the national average (as derived from the spatiotemporal random effects) was reported in 140/205 sub-districts, indicating a high rate of transmission in this part of the country. Whereas 25/205 sub-districts had > 95% probability of a trend below the national average (Fig. 4).

## Discussion

In this study, a Bayesian statistical framework was deployed to determine the climatic variability and spatio-temporal patterns of the common cold in Bhutan. The model containing both structured and unstructured random effects and the spatio-temporal random effects was the best-fitting and most parsimonious model as determined by the lowest DIC. The incidence was spatially and temporally heterogeneous with most subdistricts showing an increasing trend compared to the national trend. The risk of the common cold was higher in people aged < 15 years and females, positively associated with maximum temperature and rainfall, and negatively associated with elevation and relative humidity.

The common cold cases were spatially clustered in Mewang (under Thimphu District) and neighbouring subdistricts in the Western Region, probably related to similar geographical landscapes, and climatic and socio-demographic characteristics. Mewang subdistrict had the highest SMR and posterior mean RR that were significantly higher than the national average. The capital city, Thimphu, located in Mewang subdistrict, has the highest population density with 67.1 persons per square kilometre. The city has a wide range of potentially crowded settings such as shopping centers, schools, monasteries, entertainment and drinking venues that increase the risk of exposure to respiratory pathogens^28^. In addition to this sub-district, the model also showed an upward trend of 139 other sub-districts, which can be partly explained by the burgeoning developmental activities, construction industries and rise in the number of vehicles. This is evident in the sub-districts of Wangdiphodrang, Trongsa and Trashiyangtse, where the construction of major hydropower projects are underway. As a result, air pollution is a growing problem, which is known to be an important cause of respiratory infection^29^. The increasing trend in most of the sub-districts suggests that common cold will continue to be an important disease in Bhutan.

The incidence of common cold varied according to age, where the transmission was more pronounced in the younger population. A plausible explanation of high incidence in the younger age group might be due to their vulnerability to initial exposures of different respiratory pathogens, with risk decreasing with age as people become immune to homotypic infections. In a study carried out in children < 18 months of age, the incidence of upper respiratory tract infection was shown to increase up to one year of age and then start to decrease^30^. The incidence of viral pathogens was also found to be higher in younger age groups and then dramatically decrease with increasing age^30,31^. The other factors such as household air pollution, inadequate access to safe drinking water, and poor sanitation and hygiene might have also played a role in the spread of respiratory infections in children^29,32^. This was apparent with an escalated SMR associated with common cold in Thedtsho sub-district in Wangdiphodrang, which also had a low coverage of safe drinking water and flush toilets^33^.

Our study revealed that males were less likely to contract cold than females. Although there is no clear explanation, we speculate that differences in women’s behaviour and lifestyle (in particular indoors and peridomestic activities) might have accounted for this association. Past studies have shown mixed results with respect to sex^30,31^. As in most Asian countries, women in Bhutan undertake domestic or unpaid works, such as housekeeping, teaching children, caring sick people, cooking, dishwashing and laundry^34^. Having frequent contact with children and caring for sick people had been known to expose women to illness^35^. In addition, firewood is the common fuel used for cooking and heating in peri-urban and rural Bhutan, this could also contribute to respiratory illnesses such as common cold^36,37^. Improving methods of cooking in rural Bhutan can help address this issue and women’s and children’s health.

Our time series analysis showed two distinct peaks possibly associated with the prevalence of high frequency of respiratory pathogens circulating in the country and variation in climatic variables. The latest lists of pathogens causing respiratory infections in Bhutan have included influenza virus, parainfluenza virus, respiratory syncytial virus (RSV), rhinovirus, coronavirus, human metapneumovirus (hMPV), enterovirus, adenovirus and parainfluenza viruses^38,39^. Adenovirus, hMPV and rhinovirus are known to occur throughout the year^18,40^, while enteroviruses most commonly occur at higher temperatures, and some of these viruses are therefore known as summer viruses^18^. The current study revealed two peaks of incidence in February–April and July–September, which corresponded with the increasing influenza activity in Bhutan^41^. Additionally, in Bhutan, February is a cooler and drier month that marks the nationwide annual opening of the schools and colleges, and July coincides with the hot and monsoon season where the schools and colleges are re-opened after a midterm break. In a congregate setting like schools and colleges, introduction of viral infections by some infected students or teachers is highly likely, and thereafter spread to a large crowd. Other studies also reported a surge in respiratory infections following the returning of students to the schools after holidays^42,43^. In Israel, the infection reaches its highest rate following two weeks of opening the school^43^. This information would be useful for the policy makers to strengthen their interventions during these critical periods to reduce burden of common cold.

The current study demonstrated a significant increase of colds due to increase in temperature. An increase in temperature lowers the respiratory functions^44^, causes airways and systemic inflammation^45^, and may shift the blood flow away from vital organs to subcutaneous areas which increases stress on the lungs leading to the development of respiratory symptoms^46^. Similar to our study, previous study in China^21^ and Vietnam^47^ also found positive association between temperature and common colds. On the contrary, variations were observed in other studies^24^, where the common colds occurred more often in cold temperatures. This indicates the importance of different local climatic conditions in the pathogenesis of common colds.

Many studies in the past have also reported a high number of common cold cases^48,49^ and other diseases^50–52^ during the monsoon season which was also noted in this study. During rainy seasons, it is plausible that many people are restricted to homes where they gather together, during which time infections can spread quickly. Another possible reason for an increase in cases following rainfall might be due to contamination of surface water, which is the predominant source of drinking water in the country. Contaminated water and poor sanitation have been associated with high rates of respiratory infections^52,53^. An increase in cases of respiratory infections in the aftermath of monsoon flooding has been quite common^54^. This could be due to increased RSV, which occurs during the monsoon season^49^.

An inverse association was observed between the common cold and relative humidity. This can be partly explained by the higher efficiency of respiratory pathogen transmission at low relative humidity than higher relative humidity as evident from animal studies^18^. Enveloped respiratory pathogens such as influenza and RSV have decreased survival in places with high relative humidity^55,56^.

### Limitations

This study is subjected to a number of limitations. Firstly, we used passive notification data from the MoH database. It is likely to be under-reported if people did not seek care from the public health facilities, and therefore all cases would not have been captured by the national common cold database. However, unusually compared to other countries, reporting of colds is mandatory and this will have been likely to boost reporting rates. Secondly, other unmeasured covariates such as air pollutants, poverty index, nutritional data and access to safe drinking water were unaccounted for in this study due to the lack of data. Thirdly, since the common cold and climatic variables were aggregated at the sub-district level, the effect estimates may not reflect the biologic effect at the individual level. However, we used a fine spatial resolution to make exposure homogenous. The main strengths of this study are using a long time series data at monthly interval (108 time points) and at a fine spatial resolution (sub-districts). This would have captured local specific variation of the risk for the common cold.

## Conclusion

The common cold is predominantly a childhood illness that is spatially clustered across sub-districts in Bhutan. The illness was highly seasonal, associated with climatic conditions that might drive the circulation of different respiratory pathogens. Temperature and rainfall were both positively associated with the risk of common cold while elevation and relative humidity were negatively associated with colds in the country. The finding can be used to prioritize public health resources in areas and times of the year when climatic variables are associated with the transmission of common cold.

## Methods

### Study setting

**Figure 5:**
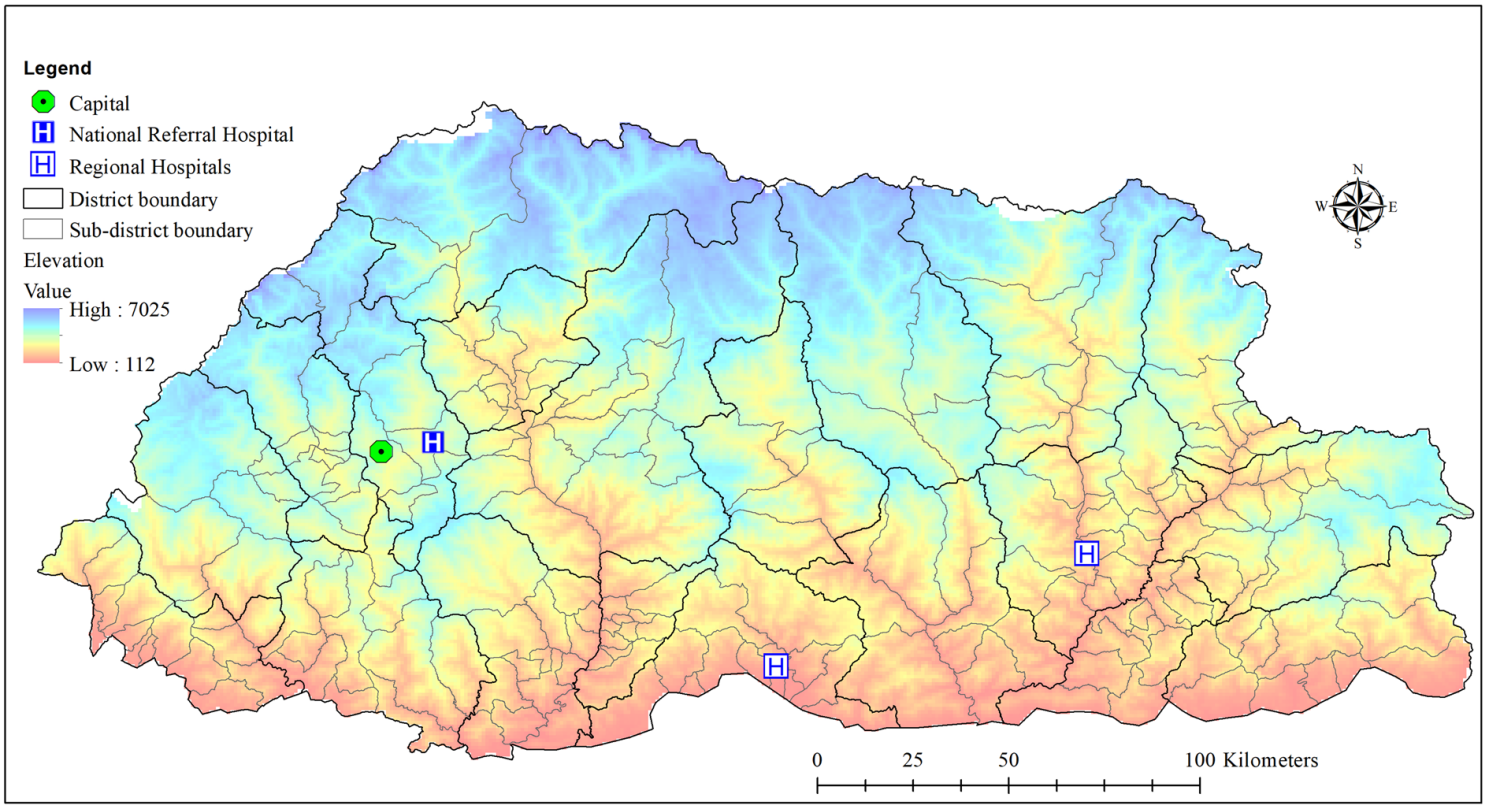
Bhutan map showing district and sub-district boundaries, capital, Paro international airport, elevation, regional and national referral hospitals.

This study was conducted in the Kingdom of Bhutan. The country is situated in the Eastern Himalayas located between China in the north, and India in the south, west and east. The altitude varies from 112 m above sea level (masl) in the southern foothills to 7025 masl in the norther parts of Bhutan. The climate is humid and subtropical in the southern plains and foothills, temperate in the inner valleys of the central region and cold in the northern Himalayas. Bhutan has an area of 38,394 km^2^ and is administratively divided into 20 districts or *dzongkhags* and 205 sub-districts or *gewogs*^33^. For this study, we used all sub-districts as the unit of spatial analysis (Fig. 5).

### Study design and data sources

This was an ecological study using secondary data collected between January 2010 and December 2018. Ecological study involves analysis of exposure at the population or group level, rather than individual level^57^. Monthly aggregated numbers of cases of cold stratified by age (< 15 and ≥ 15 years), sex and sub-district of cases were obtained from the District Health Information System (DHIS2) maintained by the Health Management and Information System (HMIS), Ministry of Health, Bhutan. The reporting of cases of cold to the HMIS through the DHIS2 is mandatory by all levels of healthcare including national and district hospitals and primary health care units (PHCs). Data from DHIS2 is used to monitor and manage patient health, improve disease surveillance and track the performance of program-specific indicators.

Over-the-counter medications for common cold are commonly reported^58–62^. However, this is likely to be less in Bhutan. Firstly, health care is free and people can get a free walk in consultation^63,64^. Therefore, people are more likely to attend hospitals and public health facilities than take medications over-the-counter. Secondly, there are no private health practitioners, so all people have to seek health care from public health facilities. Third, there are limited private drug stores in the country, mostly in the district headquarters and capital city. Therefore, over-the-counter treatment for common cold should be minimal.

Data on climatic variables (rainfall, relative humidity, and minimum and maximum temperature) were obtained from the National Centre for Hydrology and Meteorology, the Royal Government of Bhutan. Altitude was obtained from the DIVA-GIS website. Population data were obtained from the Population and Housing Census of Bhutan (PHCB), 2017, National Statistical Bureau, the Royal Government of Bhutan. The shapefiles of the sub-districts were downloaded from an open-source, the DIVA-GIS website^65^.

### Cumulative incidence and standardized morbidity ratios

The cumulative annual incidence of the common cold in each sub-district was computed for each year by age group. The overall crude standardised morbidity ratios (SMR) were calculated for each sub-district using the following formula^66^:

$${Y}_{i}=\frac{{O}_{i}}{{E}_{i}},$$

where, *Y*_*i*_ is the overall SMR for the *i*th sub-district, *O*_*i*_ is the number of observed common cold cases in the *i*th sub-district, and *E*_*i*_ is the expected number of common cold cases in the *i*th sub-district. Here, the expected number of cases was calculated by multiplying the national incidence of common cold by the average population of the sub-district during the study period.

### Seasonal patterns

The monthly mean number of common cold cases was calculated for the full time series (January 2010 to December 2018). This time series was then decomposed using locally-weighted regression or loess into three temporal components: seasonality, trend and residual variability. The model was structured with the following formula^67^:

$${Y}_{t} = {T}_{t}+ {S}_{t}+ {R}_{t},$$

where, *Y*_*t*_ is the number of cases of common cold in logarithmic transformation, *T*_*t*_, the trend component, *S*_*t*_, the seasonal component, and, *R*_*t*_, the residual component. The function *stl* and parameter setting *periodic* were used to decompose the time series data using R studio^68^.

### Spatio-temporal analysis

Initially, we performed a Poisson regression analysis to select covariates, with the number of common cold cases as the dependent variable, and climatic variables with 0, 1, 2 and 3 month lags, as independent variables. We incorporated time lags of up to three months for climatic variables in accordance with the past study^52^. Variables with *p* < 0.05 and the lowest value of the Akaike Information Criterion (AIC) were selected for inclusion in a multivariable model^69^ (Supplementary Table S1). The collinearity of the covariates was tested using the variance inflation factor (VIF) diagnostic tool^70^, where, covariates with a VIF > 4.0 were considered to be collinear and were removed from the final model. The final model contained altitude, temperature, rainfall lagged at one month and relative humidity lagged at 3 months in addition to age and sex (Supplementary Table S2).

The number of observations containing zero counts in our dataset was 17,840/88,560 (20.2%). According to the model diagnostic criteria, Zero-inflated Poisson (ZIP) regression showed better fit over standard Poisson regression with lower AIC and BIC scores. The Vuong test also showed a significant difference between the two models (see Supplementary Table S3). Therefore, ZIP regression was undertaken using a Bayesian framework. The framework has the advantage of incorporating both covariates and spatial autocorrelation in a single model with a robust evaluation of uncertainty.

Model I contained independent variables (age, sex, altitude, maximum temperature, rainfall and relative humidity) and unstructured random effects (i.e. no spatial autocorrelation was assumed in the relative risk of the common cold); Model II contained the same independent variables as Model I in addition to spatial random effects for the sub-districts; Model III, a convolution model contained independent variables as Model I in addition to unstructured and spatial random effects; and the final Model IV was built with Model III and spatiotemporal random effects.

In the last model, the observed counts of the common cold in sub-district *i* (1,…, 205), month *j* were modelled using ZIP regression as follows:

$$P \left({Y}_{ij}= {y}_{ij}\right)= \left\{\begin{array}{c}{\omega +1(1-\omega )e}^{-\mu }, \quad {y}_{ij} =0\\ {\left(1-\omega \right)e}^{-\mu }{\mu }_{ij}^{yij}/{y}_{ij}, \quad {y}_{ij} >0;\end{array}\right.$$

$${Y}_{ij}\sim Poisson\left({\mu }_{ij}\right),$$

$$\mathrm{log}\left({\mu }_{ij}\right)=\mathrm{log}\left({E}_{i}\right)+{\theta }_{ij},$$

$${\theta }_{ij}=\alpha +{\beta }_{1}Age+{\beta }_{2}Sex+{\beta }_{3}Trend+{\beta }_{4}Rainfall+{\beta }_{5}Humidity+{\beta }_{6}Altitude+{u}_{i}+{v}_{ij}+ {\mathrm{w}}_{ij},$$

where *Y*_*ij*_ is the number of common cold cases in *i* = 1,…., 205 sub-districts and month *j*; $\omega$ indicates inflation of zeros, ${\mu }_{ij}$ is the mean case, *E*_*i*_ is the expected number of cases (acting as an offset to control for population size); *θ*_*ij*_ is the log relative risk of common cold; *α* is the intercept; *β*_*1*_, *β*_*2*_, *β*_*3*_, *β*_*4*_, *β*_*5*_ and *β*_*6*_ are the coefficients for age (≥ 15 years as the reference category and assigned 0), sex (female as the reference category and assigned 0), monthly trend, rainfall lagged at one month, maximum temperature (°C) without lag, relative humidity lagged at 3 months and altitude respectively; *u*_*i*_ are unstructured random effects with a zero mean and variance *σ*_*u*_^2^, *v*_*i*_ are spatially structured random effect with a zero mean and variance σ_v_^2^ and w_*ij*_ are the spatiotemporal random effect with a mean of zero and variance of σ_w_^2^.

A conditional autoregressive (CAR) prior structure was used to model the spatially structured and spatiotemporal random effects. A queen contiguity-based spatial weight was used between sub-districts, with a weight of “1” for sub-districts with common border and “0” if they did not share a border. A flat prior distribution was specified for the intercept and a normal prior distribution for the random effects (mean of “0” and precision, the inverse of variance, set at 0.0001). The priors for the precision of unstructured, structured and spatiotemporal random effects (inverses of variances shown above), were specified using non-informative gamma distributions with shape and scale parameters equal to 0.001.

For each model, the first 10,000 burn-in iterations and convergence was assessed by visualisation of posterior density and history plots. Convergence of model parameters was checked after every 20,000 subsequent iteration blocks. All models converged at ~ 100,000 iterations. The posterior distributions of each model parameter were analysed using posterior means and 95% credible intervals (CrI). The model with the lowest deviance information criterion (DIC) was selected as the best-fit model.

In all the analyses, statistical significance was achieved with an $\alpha$-level of 0.05. The Bayesian models were developed using the WinBUGS statistical software version 1.4.3 (Medical Research Council, Cambridge, UK)^71^. Choropleth maps of SMR and the posterior random effects were produced using ArcGIS version 10.5^72^.

### Ethical considerations

Ethical clearance for this study was approved by the Research Ethics Board of Health (REBH), Ministry of Health, Bhutan (REBH/Approval/2021/105). We used secondary data for our study (from DHIS2) and there was no direct involvement of patients. As a result of this, no ethical parameters were necessary. All the methods were performed in accordance with the relevant guidelines and regulations.

## Supplementary Information

Supplementary Information.

## Data Availability

The dataset for the current study are not publicly available because Bhutan Ministry of Health owns it but are available from the corresponding author on reasonable request.
