# Supplementary material for: Identifying socio-ecological drivers of common cold in Bhutan: a national surveillance data analysis
Source: Sci Rep. 2022 Jul 9;12:11716. doi: 10.1038/s41598-022-16069-7 (PMC9271089; doi:10.1038/s41598-022-16069-7)
Supplement: Supplementary file 1 — Supplementary Information. [file 41598_2022_16069_MOESM1_ESM.pdf]

Supplementary Tables

Table S1. Univariate preliminary analysis of variables using Poisson regression

Variables	IRR	95% CI	p value	AIC
**Without lag**				
Rainfall (mm)	1.016	1.015 - 1.016	< 0.001	4382534.00
Max Temperature (⁰C)	1.044	1.044 - 1.045	< 0.001	**4325798.00**
Min Temperature (⁰C)	1.009	1.008 - 1.009	< 0.001	**4377657.00**
Relative humidity (%)	0.994	0.993 - 0.994	< 0.001	4404782.00
Altitude (masl)	1.000	0.999 - 1.000	< 0.001	4387336.00
**One month lag**				
Rainfall (mm)	1.016	1.016 - 1.016	< 0.001	**4379680.00**
Max Temperature (⁰C)	1.036	1.035 - 1.036	< 0.001	4366308.00
Min Temperature (⁰C)	1.008	1.008 - 1.008	< 0.001	4386719.00
Relative humidity (%)	0.993	0.993 - 0.994	< 0.001	4403684.00
**Two months lag**				
Rainfall (mm)	1.014	1.013 - 1.014	< 0.001	4402784.00
Max Temperature (⁰C)	1.026	1.025 - 1.026	< 0.001	4404423.00
Min Temperature (⁰C)	1.007	1.007 - 1.007	< 0.001	4402019.00
Humidity (%)	0.993	0.992 - 0.993	< 0.001	4388088.00
**Three months lag**				
Rainfall (mm)	1.008	1.007 - 1.008	< 0.001	4435252.00
Max Temperature (⁰C)	1.019	1.018 - 1.019	< 0.001	4426225.00
Min Temperature (⁰C)	1.006	1.006 - 1.007	< 0.001	4414862.00
Relative humidity (%)	0.992	0.991 - 0.992	< 0.001	**4372491.00**

Max: Maximum; min: Minimum; mm: millimetre

Table S2. Variance inflation factor (VIF) for collinearity of the selected variables

Variable	VIF
Rainfall (mm)	1.27
Maximum Temperature (⁰C)	1.23
Altitude (meters above sea level)	1.11
Relative humidity lagged 3 months (%)	1.03
**Mean VIF**	**1.16**

Table S3. Model comparison using Akaike's Information Criterion (AIC), Bayesian Information Criterion (BIC) and Vuong test

Model	Observation	AIC	BIC	Vuong's test p-value
Poisson	22,140	4250889	4250929	< 0.001
ZIP	22,140	**3286442**	**3286490**	

Supplementary figures

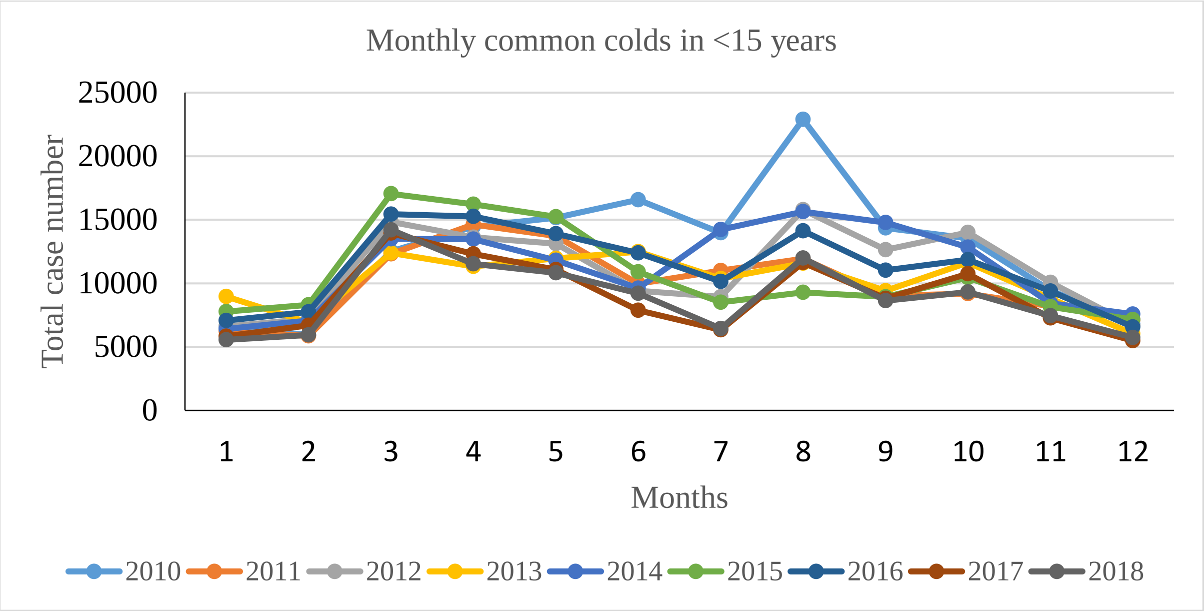

Supplementary Fig. 1 Monthly trend of common colds in < 15 years

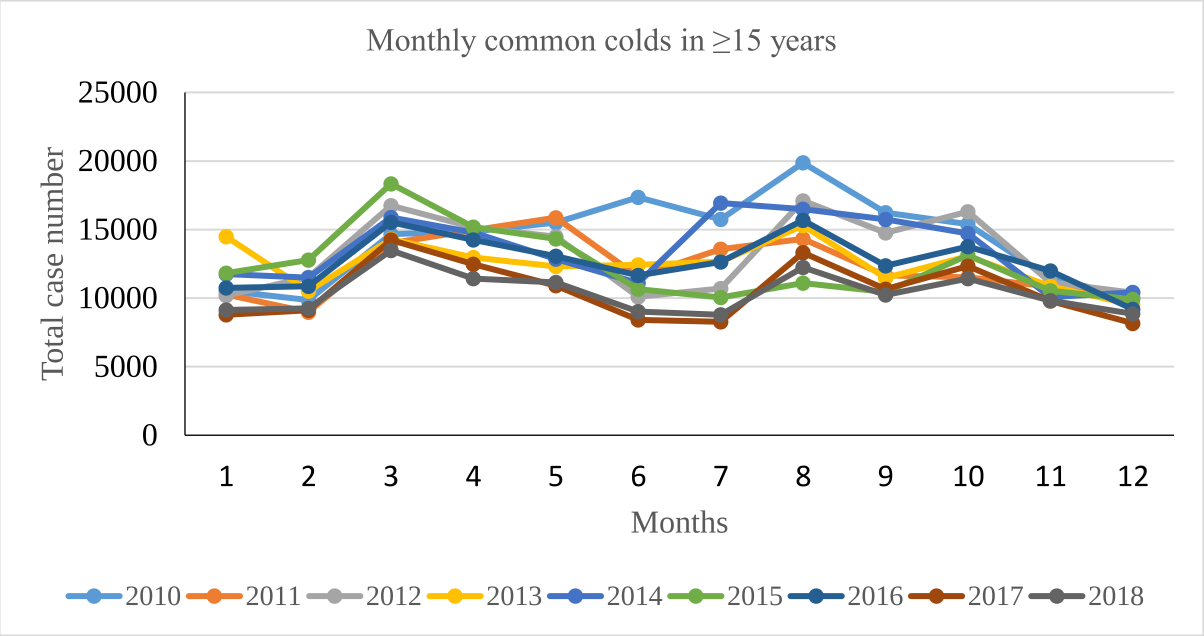

Supplementary Fig. 2 Monthly trend of common colds in ≥15 years

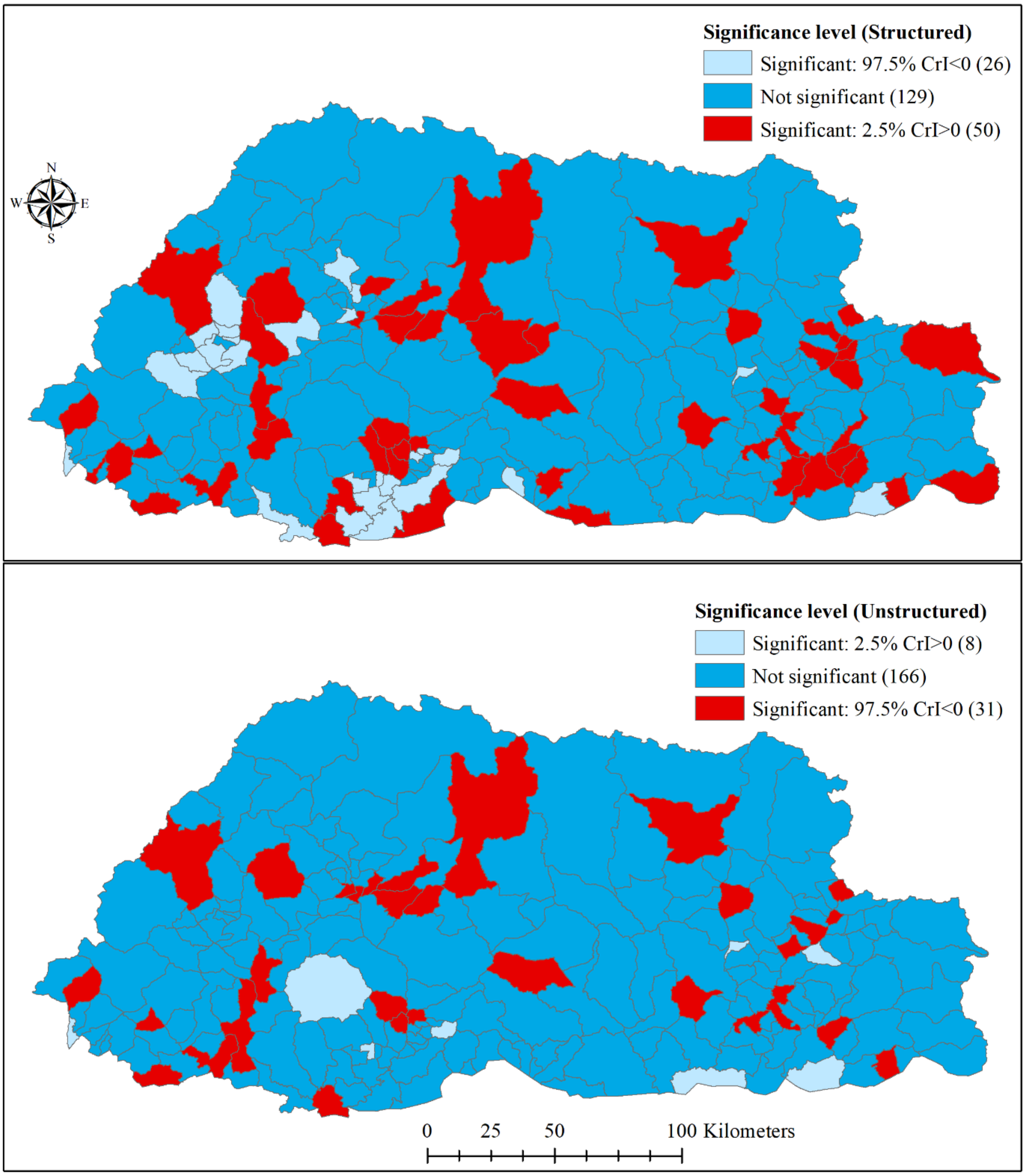

Supplementary Fig. 3 Significance map of posterior means of structured and unstructured random effect by sub-districts in Bhutan from 2010 – 2018.
